# Water Contamination Reduces the Tolerance of Coral Larvae to Thermal Stress

**DOI:** 10.1371/journal.pone.0019703

**Published:** 2011-05-11

**Authors:** Andrew P. Negri, Mia O. Hoogenboom

**Affiliations:** Australian Institute of Marine Science, Townsville, Queensland, Australia; College of Medical, Veterinary and Life Sciences, University of Glasgow, Glasgow, Scotland, United Kingdom; Institute of Marine Research, Norway

## Abstract

Coral reefs are highly susceptible to climate change, with elevated sea surface temperatures (SST) posing one of the main threats to coral survival. Successful recruitment of new colonies is important for the recovery of degraded reefs following mortality events. Coral larvae require relatively uncontaminated substratum on which to metamorphose into sessile polyps, and the increasing pollution of coastal waters therefore constitutes an additional threat to reef resilience. Here we develop and analyse a model of larval metamorphosis success for two common coral species to quantify the interactive effects of water pollution (copper contamination) and SST. We identify thresholds of temperature and pollution that prevent larval metamorphosis, and evaluate synergistic interactions between these stressors. Our analyses show that halving the concentration of Cu can protect corals from the negative effects of a 2–3°C increase in SST. These results demonstrate that effective mitigation of local impacts can reduce negative effects of global stressors.

## Introduction

Coral reefs are currently threatened by a range of anthropogenic stressors including global climate change [1] and localised pressures such as overfishing [2] and pollution [3]. Within this range of stressors, rising water temperature is generally recognised as the most immediate widespread threat to reef resilience. Under scenarios projected by the IPCC, ocean temperatures are likely to exceed the thermal thresholds of vulnerable coral species by the year 2050 [1]. The threats that climate change pose to coral reefs may be magnified by elevated levels of nutrients, sediments and pollutants from terrestrial runoff, or from point sources of pollution such as ship-groundings and mine tailings [3]. Correspondingly, government programs have been initiated to strengthen reef resilience by minimising pollution. NOAA [4] recommends that “..actions to abate impacts of fishing and land-based sources of pollution can make coral reefs more resilient to climate change..” while the Australian Federal and Queensland State Government's Reef Plan [5] aims to “halt and reverse the decline in water quality entering the Reef by 2013..” to improve the resilience of the Great Barrier Reef (GBR) to effects of climate change. Nevertheless, empirical evidence to support these policies is lacking as few studies have examined the combined, and potentially interactive, effects of climate change and pollution.

Increased SST and pollution can both impact upon corals during the vulnerable early stages of their development. Most corals reproduce by broadcast spawning, where eggs are externally fertilised and larvae develop and disperse in the water column for between 2 and ∼100 days before metamorphosing into sessile polyps [6], [7], [8], [9]. Coral spawning usually occurs during warmer months at SSTs in the range of 28–30°C throughout reef environments [10]. Depending on coral species identity, reef location and the duration of the larval dispersal period, these temperatures can be close to (within 2 to 4°C of) the thermal thresholds identified for coral larval settlement [11] and bleaching in adult colonies [12], [13], leaving limited scope for the early life stages of sensitive coral species to avoid the projected impacts of climate change.

Trace metal contamination of coral reefs from agricultural runoff, shipping accidents and operations, mining, and dredging is well recognised [14], [15], [16]. Copper (Cu) occurs naturally in the marine environment and is an essential trace element for all life; however, redox-active Cu ions can become toxic if they occur at concentrations above physiological thresholds [17]. In adult corals, Cu affects both the host tissue and symbiotic algae, reducing photosynthesis [18] and triggering the breakdown of symbiosis [19]. Despite this sensitivity of adult corals, it is during the recruitment phase of the coral life-cycle that pollution by metals and organometallic compounds can pose the greatest risk [14]. Laboratory experiments have shown that successful metamorphosis requires relatively uncontaminated surfaces for coral larvae to attach to as they transform into sessile polyps, and that Cu inhibits this metamorphosis at lower concentrations than any metal tested so far [20], [21].

Temperature exerts control over metabolism and biochemistry and may therefore enhance, or counteract, the toxicity of pollutants [22]. Whether or not these two stressors interact is especially relevant to ectothermic and sessile organisms like corals, which have no control over their tissue temperature nor their exposure to pollution [23]. While many factors contribute to the dynamics of coral communities [24], recent studies indicate that recruitment is an important factor in determining the response of coral populations to bleaching and mass mortality events [25]. Despite the potential for water contamination to exacerbate the effects of temperature on coral recruitment, the relationship between temperature, pollution and metamorphosis is poorly studied. To help fill this critical knowledge gap, we conducted laboratory exposure studies to assess the response of larvae of two coral species to different combinations of temperature and pollution (Cu). The results allowed the development and implementation of a quantitative framework to assess how pollution impacts the thermal tolerance of coral larvae.

## Results and Discussion

### Functional relationship between metamorphosis success, temperature and copper

For a range of taxa, growth and/or development rates decrease when metal contamination reaches a threshold level [26], [27]. If contaminant concentrations exceed this threshold, development is increasingly inhibited and eventually declines to zero. This type of functional response can be generally characterised by a 3-parameter sigmoid equation, as:
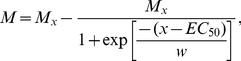
(1)where, in the case of coral larval metamorphosis, *M* is the proportion of larvae that metamorphose at a given copper (Cu) concentration (*x*), *M_x_* is the maximum achievable metamorphosis percentage, *EC_50_* is the copper concentration at which metamorphosis is half of its maximum value and *w* is proportional to the width of the region over which metamorphosis declines from *M_t_* to zero. This equation is a simplification of the 4-parameter logistic equation commonly used to model dose-responses [28] - because in this case the fourth parameter (minimum level of metamorphosis success) is zero. In the present study, a range of copper concentrations of up to 70 µg l^−1^ (above the maximum reported in the field) was used to ensure precise estimation of model parameters and full characterisation of the functional relationship between Cu and metamorphosis success. To incorporate temperature into this general model, we hypothesised that the shape of the functional relationship between metamorphosis and Cu concentration (i.e., the values of the parameters of Equation 1) depend on temperature.

**Figure 1:**
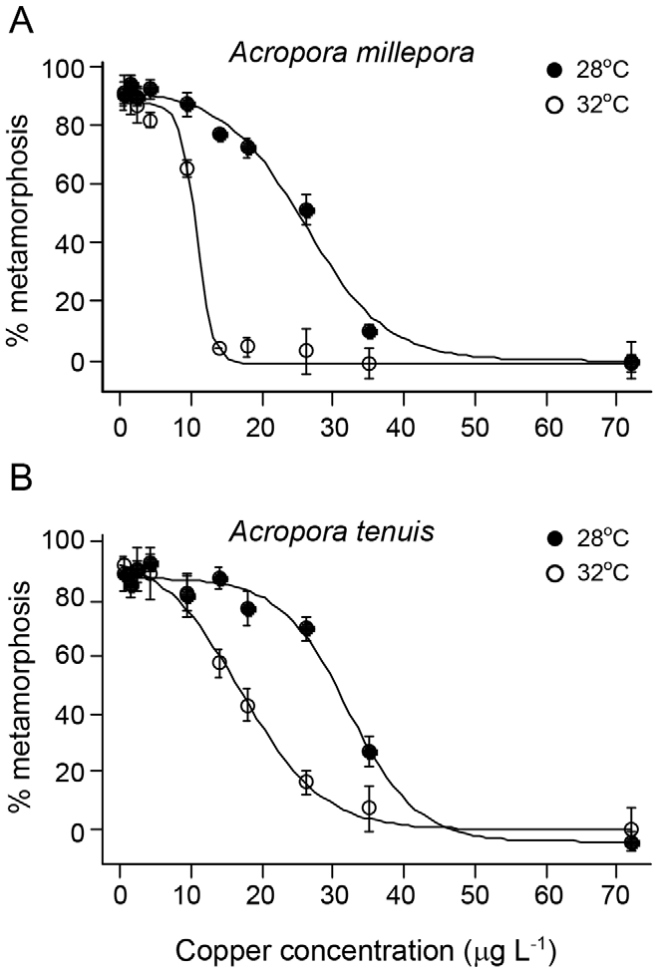
Relationship between larval metamorphosis and copper concentration for *Acropora millepora* (a) and *Acropora tenuis* (b). Points represent the mean and standard error of measured % metamorphosis at each of two temperatures. Lines are the best-fit non-linear regressions of Equation 1 to each set of data.

Metamorphosis success of aposymbiotic *Acropora millepora* and *A. tenuis* larvae was inhibited by Cu in the laboratory, and a sigmoidal relationship adequately captured the variation in the data (Equation 1, Fig. 1). In the majority of cases, the goodness-of-fit (R^2^) of Equation 1 to metamorphosis data at each temperature ranged between 0.88 and 0.94 and the estimated parameters of Equation 1 were significantly different from zero. However, at high incubation temperatures (i.e., above 33°C) the Cu-metamorphosis relationship was more variable, with 0% metamorphosis observed in a large number of the experimental replicates, and the R^2^ values for these model fits decreased to 0.57 (*A. millepora* at 33°C), 0.76 and 0.25 (*A. tenuis* at 33°C and 34°C respectively). These results demonstrate that the thermal optimum for larval metamorphosis in the laboratory was less than 32°C for both coral species, consistent with other reports that coral larvae are almost as susceptible to high SSTs as adult corals [11], [29], [30]. However, whereas adult corals are primarily susceptible to thermal stress due to the accumulation of oxygen radicals in their photosynthetic symbionts [31], most coral larvae (including *A. millepora* and *A. tenuis*) do not contain significant numbers of acquired symbionts and the cause of impaired function (metamorphosis) is unclear. Like adult corals, *Acropora* larvae infected with photosynthetic symbionts are more susceptible to thermal stress, exhibiting higher levels of antioxidant defences and oxidative cellular damage than those without symbionts [32]. A recent study revealed complex molecular responses of azooxanthellate *A. millepora* embryos and larvae to high SST [33]. In that study, the genes regulating metabolic rate (respiration) were more affected by thermal stress than those involved in protection from oxidative stress, indicating that oxidative stress may not be the primary factor affecting the success of larval settlement at high SST.

**Figure 2:**
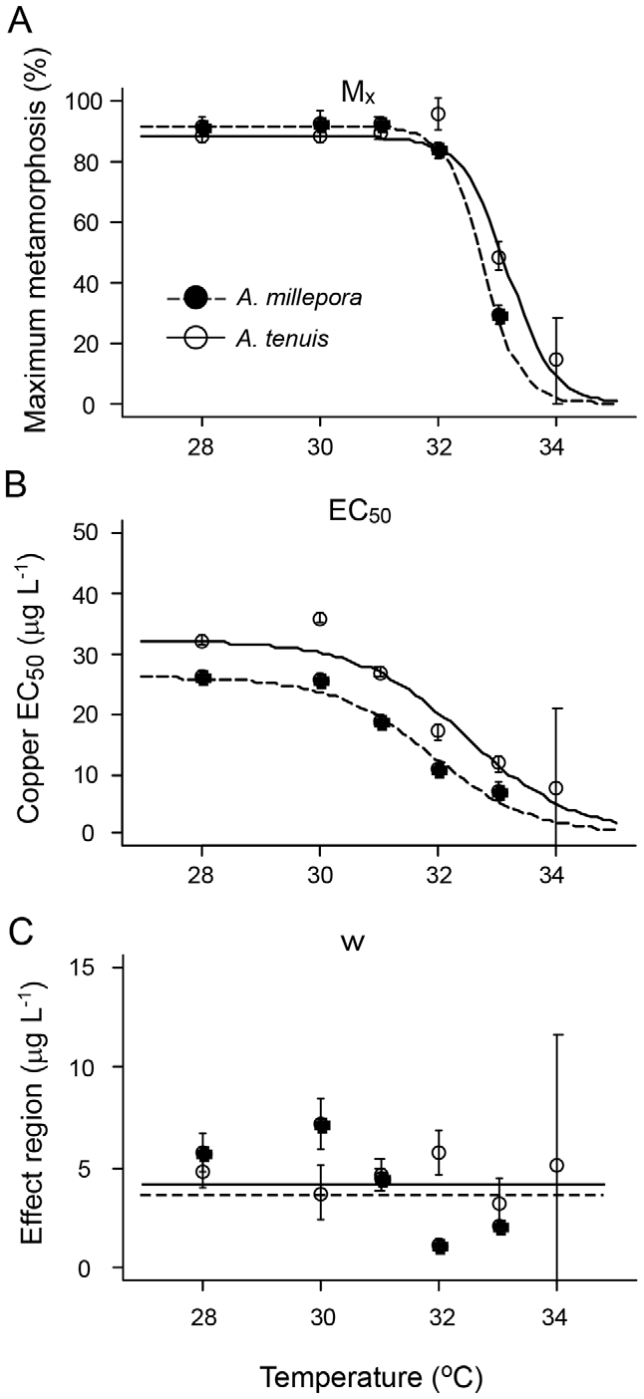
Temperature dependence of the parameters of the relationship between larval metamorphosis and copper contamination for *Acropora millepora* (filled points, dashed line) and *Acropora tenuis* (open points, solid line). Lines are fits of Equation 2 to data for each parameter and points represent the mean and standard error of the best-fit parameter estimates from the fits of Equation 1 to metamorphosis data at each temperature.

The parameters describing the shape of the relationship between Cu concentration and metamorphosis success changed clearly and consistently with temperature (Fig. 2). For both species, the parameter describing maximum metamorphosis (*M_x_*) success declined when temperatures exceeded 32°C, but was approximately constant at temperatures below this value (Fig. 2A). The Cu concentration at which metamorphosis success had decreased to half of its initial value (*EC_50_*) also decreased with temperature (Fig. 2B, Table 1). This parameter was more sensitive to temperature than *M_x_*: *EC_50_* began to decline at temperatures over 30°C. The rate at which metamorphosis success declined with increasing Cu, (indicated by the effect region parameter, *w*) did not vary consistently with temperature (Fig. 2C).

**Table 1:** Copper concentrations (EC_50_s) and temperatures (IT_50_s) that inhibit 50% metamorphosis in *Acropora millepora* and *A. tenuis.*

Level	*A. millepora*	*A. tenuis*
*Temperature*	*EC_50_ (µg l^−1^)*	*EC_50_ (µg l^−1^)*
28	26.03 (0.98)	32.10 (0.86)
30	25.60 (1.29)	35.76 (0.83)
31	18.97 (0.60)	26.86 (0.93)
32	10.67 (0.39)	16.97 (1.19)
33	7.21 (1.56)	11.79 (1.31)
34	nd	7.49 (13.44)
*Cu concentration*	*IT_50_s (°C)*	*IT_50_s (°C)*
0.37	32.77 (0.06)	33.17 (0.09)
1.43	32.73 (0.07)	34.62 (1.53)
2.37	32.72 (0.09)	33.09 (0.08)
4.13	32.69 (0.08)	32.91 (0.08)
9.4	32.23 (0.09)	32.92 (0.06)
13.8	31.52 (0.15)	32.33 (0.09)
17.8	31.56 (0.15)	32.07 (0.09)
26.2	31.27 (0.08)	31.46 (0.11)
35	nd	nd
72	nd	nd

Standard error in brackets.

**Figure 3:**
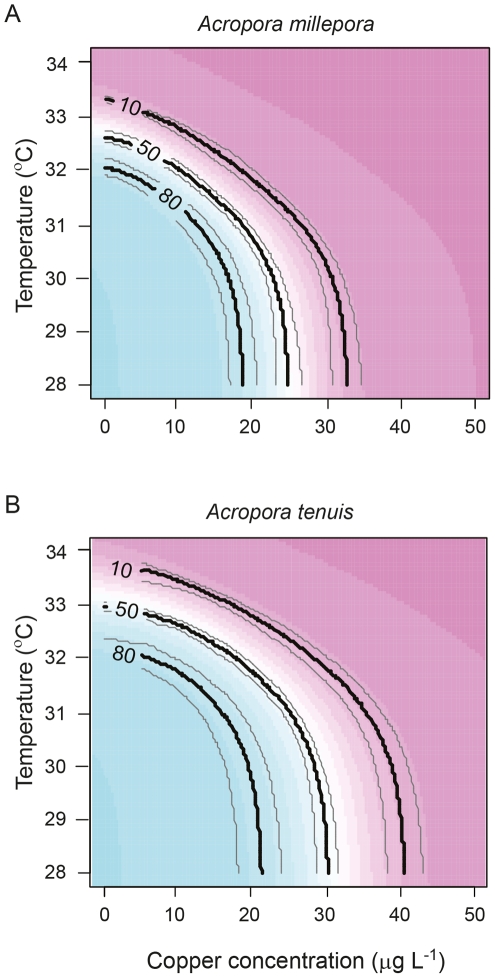
Environmental thresholds for larval metamorphosis of *A. millepora* (a) and *A. tenuis* (b). Color depicts the change in metamorphosis success from >80% (blue) to 0% (pink) with increasing copper concentration and temperature. Thick lines show 80%, 50% and 10% metamorphosis contours based on the best-fit parameter estimates (Table S2) and thin, gray lines depict the 95% CI around each threshold. CI's were generated from a Monte Carlo simulation technique where the model was iterated 1000 times using parameters randomly drawn from multivariate Gaussian distributions.

The completed analysis resulted in a fully parameterised model of larval metamorphosis success incorporating both the Cu and temperature responses. A strength of this approach is that it allows full characterisation of thresholds for larval metamorphosis success together with a robust quantification of how sensitive these thresholds are to uncertainty in parameter estimates. Metamorphosis thresholds were identified by evaluating the fitted model for each species over a range of temperature and Cu concentrations, and plotting contours lines corresponding to 0%, 10%, 50% and 80% metamorphosis (Fig. 3). The temperature and Cu values corresponding to each metamorphosis threshold were robust with respect to the uncertainty in parameter estimates. The 95% confidence interval (thin gray lines in Fig. 3) around the metamorphosis thresholds were narrow and parallel to the threshold based on the (mean) best-fit parameter values. In general, larval metamorphosis success of both *Acropora millepora* and *A. tenuis* was similarly inhibited by temperature and Cu contamination (Fig. 2, Fig. 3). However, *A. tenuis* larvae were slightly more resistant to both variables: the *EC_50_* value was higher at all temperatures for *A. tenuis* (Fig. 2B, Table 1) and metamorphosis thresholds were broader for this species (contour lines in Fig. 3). The full Cu and temperature model demonstrates that, if seawater Cu concentrations are maintained below 15 µg l^−1^, maximum metamorphosis success is greater than 80% at temperatures up to 32°C (Fig. 3). However, an increase in Cu concentration to 30 µg l^−1^ reduces maximum metamorphosis success for *A. millepora* to 20%, with a subsequent decrease in metamorphosis by 5% for every 1°C increase in temperature (Fig. 3A). For *A. tenuis* at the same Cu concentration, maximum metamorphosis success was 50% and decreased, on average, by 10% for every 1°C increase in temperature (Fig. 3B). The Cu thresholds identified in these laboratory exposures lay within the range of values reported for oceanic waters. At background environmental levels, Cu concentrations in seawater are generally less than 1 µg l^−1^. Nevertheless, concentrations of 5 µg l^−1^ have been reported in tropical coastal waters [34], with the highest concentration reported reaching 30 µg l^−1^ [35]. While runoff from mine tailings, industry and urban sources can introduce metals such as Cu into reef ecosystems, ship groundings are a direct source of extreme Cu concentrations on coral reefs (up to 4270 µg g^−1^ sediment) due to the antifouling paint containing Cu being crushed onto the reef structure [36], [37].

### Evaluating synergism between temperature and copper

**Figure 4:**
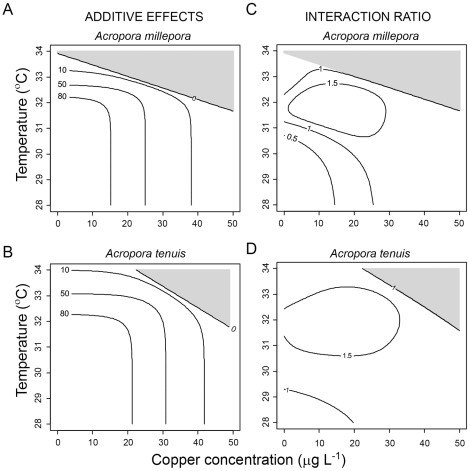
Theoretical additive effects (a, b) of temperature and copper on larval metamorphosis compared with observed interactive effects (interaction ratio, *IR*) of these factors (c, d). Additivity was calculated as the sum of the effect of copper at the control temperature and the effect of temperature at the control (lowest) copper concentration and contours represent metamorphosis success. *IR* was calculated from Equation 3: values >1 demonstrate synergy and values <1 demonstrate antagonism. Shaded regions depict combinations of copper and temperature for which metamorphosis success was zero.

The shape of the relationship between metamorphosis success and Cu concentration was strongly influenced by temperature (Fig. 1, Fig. 2). Two-way ANOVAs indicated significant interactions between the effects of temperature and Cu concentration on metamorphosis success for both coral species (Table 2), signifying that overall, the combined effects of these stressors were not additive. The effects of SST and Cu on metamorphosis success were strongly non-linear making ANOVA inappropriate for detection of the nature of the interaction [38]. However the modelling technique and isobologram analysis employed here were effective tools for identifying regions of the parameter space which gave rise to synergistic versus antagonistic interactions. The model was used to calculate expected 10%, 50% and 80% metamorphosis success contours for additivity. These contours were almost vertical up to 31°C when plotted as isobolograms for both species (Fig. 4A and 4B), indicating little or no expected effect of temperature on toxicity in this region. As temperature started to affect metamorphosis (>31°C), the additive effects of temperature and Cu concentration were expected to cause a rapid decrease in metamorphosis as Cu increased. The strength of interaction between SST and Cu was quantified by dividing the observed effect on metamorphosis by the expected additive effect from the modeled data. The interaction plot (Fig. 4C) indicated sub-additivity (interaction ratio, *IR*<1) at low temperature-Cu combinations for *A. millepora*, increasing to additive effects at temperatures less than 31°C and then becoming strongly synergistic (*IR*>1) at temperatures between 31°C and 33°C and Cu concentrations up to 30 µg l^−1^. The response of *A. tenuis* was similar; however, there was little apparent sub-additivity and the range of temperature and Cu concentrations where metamorphosis was reduced by 50% more than expected for additivity (*IR* = 1.5) was broader for this species (Fig. 4d). Overall, Cu contamination and temperature stress had a stronger synergistic effect in inhibiting metamorphosis of *A. tenuis* compared to *A. millepora*, although the latter species was generally more sensitive to these stressors. Three other studies have examined the combined effects of SST and pollution on adult corals but these used fewer treatment combinations, or a narrower range of treatments, making interactions more difficult to quantify. Nystrom et al. [39] found that the combination of elevated SST (ambient and 4°C above ambient) and Cu (0 and 11 µg L^−1^) did not interact to affect coral metabolism. Two other studies found that the effect of the herbicides on photosynthesis of coral symbionts decreased as temperature increased from 26 to 30°C, indicative of an antagonistic interaction [40], [41]. Nevertheless, the latter study also showed that two herbicides acted synergistically with higher SSTs (31 and 32°C) as demonstrated by a greater than additive effect on inhibition of symbiont photosynthesis [41]. The current study is the first to demonstrate synergistic effects of environmentally relevant SST and levels of pollution that directly affect corals (rather than their symbionts) at a critical phase during their life-histories.

**Table 2:** Summary of analysis of variance testing the mean effects of experimental copper and temperature treatments on larval metamorphosis for *Acropora millepora* and *Acropora tenuis*.

Factor	Sum of squares	df	Mean Square	F ratio	p value
*Acropora millepora*					
Temperature	55	5	10	365	<0.001
Copper	43	9	4.7	168	<0.001
Temperature × Copper	18	45	0.39	14	<0.001
Residual	8.4	300	0.03		
*Acropora tenuis*					
Temperature	45	5	9.1	285	<0.001
Copper	43	9	4.7	148	<0.001
Temperature × Copper	10	45	0.23	7.1	<0.001
Residual	9.6	300	0.03		

### Implications of climate and pollution interactions for coral recruitment

The results from this study demonstrate that the critical early life stages of coral development, during which corals metamorphose from pelagic larvae into sessile polyps, are more sensitive to high SSTs in the presence of the common anthropogenic pollutant copper (Cu). We found evidence of strong interactions between Cu and temperature as determinants of metamorphosis success for two common coral species. The combined effects of Cu and increased SST were additive for *A. tenuis* larvae above 29°C and became synergistic for both species at sea surface temperatures (SSTs) above 31°C, with the combined effect of Cu contamination and excessive SST being stronger than the sum of the independent effects of each stressor. These findings provide empirical evidence to support the implementation of environmental policies that aim to increase the resilience of corals to elevated SSTs by improving water quality. Although synergistic effects of these two stressors were only apparent at temperatures above those which would be presently experienced by the majority of settling larvae on the GBR, additive effects of Cu and temperature were apparent at ecologically relevant temperatures.

Molecular techniques indicate that some of the sub-cellular responses of corals to Cu may be similar to those identified for thermal stress. Heat-shock proteins (Hsp 70 and Hsp 90), normally associated with thermal stress, were up-regulated in adult *Montastraea franksi* branches exposed to Cu during an 8 h period [42]. While shared stress response pathways may be partially responsible for the interactive effects of increased SST and Cu on metamorphosis, the effects of temperature on larval metabolism or biochemistry may also influence the toxicity of Cu [22]. Uptake of Cu by the larvae can increase with temperature due to: (i) increased active transport (facilitated or via ion channels), (ii) increased membrane permeability, (iii) increased metabolic rate, and/or (iv) reduced Cu elimination [23]. Furthermore, each of these mechanisms can be influenced by the species of Cu, which is likely to change as the SST increases. In seawater, Cu exists in equilibrium between a range of species, from the most toxic and bioavailable form of free copper (Cu^2+^, not measured in the present study) to less bioavailable carbonate complexes [43], [44]. The present study demonstrates that coral larval metamorphosis, a critical step in the process of coral recruitment, is more sensitive to Cu as SSTs increase further above current summer temperatures of ≥31°C [45]. Copper affected metamorphosis at concentrations close to or below the US EPA guideline figure of 3.1 µg L^−1^ [46], at temperatures ≥32°C. For instance, in addition to the effect of temperature Cu concentrations of approximately 4.4 µg L^−1^ and 0.4 µg L^−1^ lead to a further 10% reduction in metamorphosis success for *Acropora millepora* larvae at 32°C and 33°C respectively, and Cu concentrations of approximately 14.8 µg L^−1^ and 10.4 µg L^−1^ caused the same effect for *A. tenuis* larvae at these temperatures (Equation 1 and 2). In fact, *Acropora* larvae tested in this study were as susceptible to Cu at 32°C deg as *Mytilus edulis* embryos, the most sensitive marine organisms previously reported [46].

The monsoon season poses the greatest challenge to the survival of nearshore coral communities [3]. During this period heavy rains transport sediments from land to sea bringing the highest levels of water contamination that are experienced over the annual cycle [3]. Coral spawning typically occurs after the full moon in the month before maximum rainfall and can coincide with high SSTs [47], [48]. The average monthly temperature (October – November between 2000–2010) during coral spawning in Nelly Bay where the corals for this study were collected ranged from 26.3 to 29.8°C [45]. However, synergistic interactions between temperature and Cu occurred in the present study at and above 31°C for both species (Fig. 4C, 4D) indicating that greater than additive effects of these stressors are presently unlikely to be experienced at this site except for that proportion of larvae that show delayed metamorphosis. Peak rainfall in the GBR catchments is from December to March [49], but early rainfall of the monsoon season in November and December may also deliver the high loads of pollution into the GBR lagoon [3]. Although SSTs thresholds for coral larvae and the highest loads of river-borne pollution do not usually coincide with the major coral spawning events, they are often only a month apart and this may become more of a concern as climate change impacts upon SST and rainfall patterns in the tropics. SSTs are projected to increase by between 1.8–4.0°C by 2100 (IPCC 2007), potentially impacting upon the success of coral recruitment, especially if pollution thresholds are exceeded. Furthermore, some corals do spawn during the peak SST and rainfall months of December to February [50] and these late-spawning species and/or colonies may be at greater risk from the combined effects of high SST and pollution from flood plumes. To quantify this risk, both field studies (where unknown factors may impact upon the interpretation of observations) and laboratory approaches (where stress-response relationships can be precisely described – but interpretations may be oversimplified due to the absence of other environmental influences) are needed to understand the combined effects of climate change and pollution. A recent field study indicated that wastewater discharge has increased the susceptibility of coral communities in the Florida Keys to thermal bleaching [51]. Similarly, the occurrence of coral bleaching on the GBR can be more accurately predicted when dissolved inorganic nitrogen (DIN, a nutrient found in agricultural runoff) concentration is included in the modelling framework [52]. Such models indicate that reducing DIN by 50% –80% would help to protect inshore corals of the GBR by increasing the bleaching threshold by 2°C.

Successful coral recruitment is important for the maintenance and recovery of coral communities under pressure from climate change and other anthropogenic influences [25], [53]. The profound effect that Cu has on exacerbating the negative effects of thermal stress on coral larval metamorphosis in the laboratory illustrates that water quality can be a particularly pressing issue for the health of coral reefs as SSTs increase due to climate change. The larval metamorphosis model developed here from experimental data demonstrates that reducing water contamination can have positive effects on coral recruitment. At a seawater temperature of 28°C, 50% of *Acropora millepora* and *A. tenuis* larvae successfully metamorphosed when Cu concentrations were approximately equal to 25 and 30 µg L^−1^ respectively (Fig. 3). However, halving Cu concentrations from these values resulted in more than 3.5°C increase in the temperature threshold for both species (Fig. 3). Indeed, for each of the percentage metamorphosis thresholds depicted in Fig. 3, halving Cu concentrations from the value corresponding to the control temperature (i.e. the x-intercepts of each contour line) led to an increase in the temperature tolerance by 3–5°C. This study therefore provides empirical evidence to support government programs [4], [5] that aim to improve water quality to mitigate the negative effects of increasing seawater temperatures due to global change.

## Materials and Methods

### Coral collection and larval cultivation

A total of six gravid colonies of *A. millepora* and *A. tenuis* were collected from 3 to 5 m depths near Magnetic Island, a nearshore, high-turbidity site off the north east cost of Queensland, Australia (19°10′ S, 146°52′ E). These colonies were maintained in flow-through outdoor aquaria (27°C) at the Australian Institute of Marine Science (Townsville, Australia) until coral spawning on the 29^th^ (*A. tenuis*) and 30^th^ (*A. millepora*) of October 2007. The gametes were collected and the azooxanthellate larvae cultured in indoor flow-through aquaria (27°C) using methods described in [54]. Copper solutions and analysis

Stock Cu solutions were prepared individually in MilliQ water using CuCl_2_ (Sigma). Secondary stock solutions (10× higher than final concentrations) were then prepared in 0.45 µm filtered seawater (FSW) and pHs adjusted to 8.2 with NaOH. Identical solutions were prepared and sampled for Cu analysis at Charles Darwin University by ICP- MS (Agilent 750ce) using a seawater standard additions method. Quality control for the analysis included NASS-5 and CASS-4 Certified Reference Materials, in-house reference materials and spike recoveries. To control for potential differences in water quality parameters between treatments, dissolved oxygen (mg L^−1^) and pH were also measured at selected temperatures and Cu concentrations at t = 0 and t = 24h for treatments containing 10 day old *A. millepora* larvae.

### Copper exposures and metamorphosis assays

Assays of metamorphosis success at different copper and temperature exposures were conducted using 7 day old larvae of both species. Ten to fifteen larvae were transferred to 6-well cell culture plates (12 mL, Nunc, NY, USA) in a final volume of 9 mL 0.45 µm FSW at 27°C. Stock Cu solutions (1 mL, prepared as described above) were added to individual wells to final nominal concentrations of 0, 1, 2, 4, 8, 12, 16, 24, 32 and 64 µg l^−1^. The solutions were then transferred into incubators set at 27, 30, 31, 32, 33 and 34°C (range ±0.2°C) and 30 µmol quanta m^−2^ s^−1^. Six replicate wells were used for each of the 60 treatments (10 Cu concentrations ×6 temperatures). The larvae were pre-exposed to the elevated temperatures and/or Cu for 6 h. After this period, larval metamorphosis was initiated by the addition of a slightly sub-optimal concentration (10 µL) of crustose coralline algae extract to maximise the sensitivity of the assay [55]. This extract was prepared by extracting 4 g of the crustose coralline algae *Neogoniolithon fosliei* and *Porolithon onkodes* with methanol according to the methods of Heyward and Negri [56]. After a further 18 h, treatments were terminated by adding 0.5 mL fixative (4% formaldehyde buffered at pH 7) for later assessment of settlement and metamorphosis. In addition, identical larval exposures and assays were performed in each of the incubators using 9 day old *A. millepora* larvae to test for differences in metamorphosis due to the incubators alone (Cu = 0, 16 and 32 µg L^−1^ at 31°C, the median temperature in the range tested). Water quality parameters were generally consistent across the experimental treatments. There was good correspondence between nominal and measured Cu concentrations (Table S1). Dissolved oxygen (DO) concentrations were generally between 5.9 and 6.6 mg l^−1^ and pH was approximately constant across experimental trials (8.1 to 8.2 pH units). The only exception to this was a slight reduction in DO at 34°C (Table S1). There was no effect of the experimental incubator (F_0.01,5_ = 0.5, p = 0.78) on measured larval metamorphosis success at 31°C, nor did the effect of Cu concentration on metamorphosis vary between incubator units (F_0.13,10_ = 0.32, p = 0.97).

### Modeling effects of temperature and copper contamination on larval development

To estimate the model parameters, data from the metamorphosis assays were converted to percentage metamorphosis success and analyses were conducted using the R statistical platform [57]. In this study we characterised the combined effect of copper and temperature on metamorphosis by fitting Equation 1 to metamorphosis versus Cu concentration data at different temperatures and then analysing how the fitted parameters of the equation (*M_x_*, *EC_50_* and *w*) changed with temperature. First, we used a nonlinear regression routine (‘nls’) to fit Equation 1 to the metamorphosis versus copper data to quantify *M_x_*, *EC_50_* and *w* at each temperature. Second, we again used nls to fit a sigmoid equation to the set of temperature-specific parameter estimates, as:
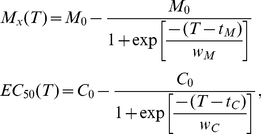
(2)where *M_x_(T)* and *EC_50_(T)* are temperature-dependent parameters of Equation 1, *M_0_* and *C_0_* are the maximum values for each of *M_x_(T)* and *EC_50_(T)* respectively, *T* is temperature, *t_M_* and *t_C_* are the temperature values at which *M_x_(T)* and *EC_50_(T)* have declined to 50% of their maximum values respectively and *w_M_* and *w_C_* are proportional to the region of temperature values over which *M_x_(T)* and *EC_50_(T)* respectively decline to zero. The values for each parameter can be found in Table S2. In this second stage of the analysis, the sigmoid equation was chosen *a posteriori* because we had no prior evidence as to how each parameter of the copper functional response would vary with temperature. To reduce the number of fitted parameters, we fixed the values of *M_0_* and *C_0_* at the values observed at 28°C (the control temperature). These particular parameters were chosen because the Cu and temperature thresholds for larval metamorphosis are strongly dependent upon the values of *t_M_, t_C_, w_M_* and *w_C_* and w, not on *M_0_* or *C_0_*. There was no relationship between temperature and the value of the parameter *w*. Therefore, all subsequent analyses used the average value of this parameter (averaged across temperatures but within species).

Uncertainty in parameter estimates was incorporated using Monte Carlo simulation. To do this we iterated the model 1000 times using parameters randomly drawn from multivariate normal distributions. These distributions were based on the variance-covariance matrices of the parameters describing the relationship between metamorphosis success and Cu concentration at each temperature. In summary, we randomly generated 1000 sets of parameter estimates for each metamorphosis versus Cu relationship (Equation 1), re-fit Equation 2 to each parameter set, and then re-evaluated the fitted model. The simulations were based on uncertainty in the Cu relationship, rather than uncertainty in the parameter temperature relationship, because the former is large relative to the latter.

Two approaches were used to determine whether there effects of temperature and Cu concentration were additive, synergistic or antagonistic. First a two-way fixed effects analysis of variance (ANOVA) was performed on the larval metamorphosis data (arcsine transformed to meet ANOVA assumptions). In this analysis, a non-significant interaction term (p temperature x Cu concentration >0.05) would demonstrate that the effects of temperature and Cu were additive whereas a significant interaction indicates the presence of a synergistic (greater than additive) or antagonistic effect (less than additive) [58]. Where an significant interaction was identified, we used an isobologram approach to determine whether the effects were synergistic or antagonistic [38]. To do this, expected metamorphosis success was calculated, across the experimental range of temperature and copper concentrations, assuming additive effects [59]. Additivity was calculated as the sum of the effect of Cu at the control temperature (28°C) and the effect of temperature at the control (lowest) Cu concentration, with these independent effects characterised from the fit of Equation 1 to these data. Subsequently, the direction and strength of the interaction between temperature and copper, across the experimental ranges, was calculated by dividing the observed (modelled) effect (% inhibition, *E_x_*) by the predicted additive effect (% inhibition, *E_p_*) as described in Equation 3. Synergy was indicated where the interaction ratio (*IR*) was >1 and antagonism (sub-additivity) where the *IR*<1 [60].



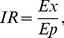
(3)


We note that the interaction ratio calculated in this study is the inverse of that in Li et al. [60] because we believe it is more intuitive that stronger than additive (i.e. synergistic interactions) have *IR* vales greater than 1.

## Supporting Information

Table S1 **Concentrations of copper, dissolved oxygen and pH in experimental treatments.**
(DOC)

Table S2 **Model fit statistics reporting overall goodness of fit of Equation 2 to the relationship between incubation temperature and the parameters describing the relationship between copper contamination and larval metamorphosis for each species.** Values represent the best-fit parameter estimates and their standard errors. All estimated parameter were significantly different from zero (p<0.05).
(DOC)
