# Supplementary material for: Water Contamination Reduces the Tolerance of Coral Larvae to Thermal Stress
Source: PLoS One. 2011 May 11;6(5):e19703. doi: 10.1371/journal.pone.0019703 (PMC3092768; doi:10.1371/journal.pone.0019703)
Supplement: Table S1 — Concentrations of copper, dissolved oxygen and pH in experimental treatments. (DOC) [file pone.0019703.s001.doc]

Table S1. Concentrations of copper, dissolved oxygen and pH in experimental treatments.

| time | Copper (µg l^-1^) | | pH | Dissolved oxygen (mg l^-1^) | | | | | |
| --- | --- | --- | --- | --- | --- | --- | --- | --- | --- |
| (h) | nominal | measured |  |  | | | | | |
|  | 31°C | 31°C | 31°C | 28°C | 30°C | 31°C | 32°C | 33°C | 34°C |
| 0 | 0 | - | 8.24 | 6.6 | 6.5 | 6.5 | 6.4 | 6.2 | 5.6 |
| 24 | 0 | 0.370 | 8.14 | 6.4 | 6.5 | 6.1 | 6.2 | 6 | 5.2 |
| 24 | 1 | 1.43 | 8.25 | - | - | - | - | - | - |
| 24 | 2 | 2.37 | 8.16 | - | - | - | - | - | - |
| 24 | 4 | 4.13 | 8.11 | 6.5 | 6.4 | 6.3 | 6.3 | 6.1 | 5.4 |
| 24 | 8 | 9.40 | 8.22 | - | - | - | - | - | - |
| 24 | 12 | 13.8 | 8.09 | - | - | - | - | - | - |
| 24 | 16 | 17.8 | 8.13 | 6.2 | 6.5 | 6.4 | 6.1 | 5.9 | 5.4 |
| 24 | 24 | 26.2 | 8.12 | - | - | - | - | - | - |
| 24 | 32 | 35.0 | 8.2 | 6.1 | 6.2 | 6.2 | 5.9 | 6 | 5.1 |
| 24 | 64 | 72.0 | 8.17 | 6.1 | 6.5 | 6.5 | 6.2 | 6.2 | 4.9 |
