# Supplementary material for: Water Contamination Reduces the Tolerance of Coral Larvae to Thermal Stress
Source: PLoS One. 2011 May 11;6(5):e19703. doi: 10.1371/journal.pone.0019703 (PMC3092768; doi:10.1371/journal.pone.0019703)
Supplement: Table S2 — Model fit statistics reporting overall goodness of fit of Equation 2 to the relationship between incubation temperature and the parameters describing the relationship between copper contamination and larval metamorphosis for each species. Values represent the best-fit parameter estimates and their standard errors. All estimated parameter were significantly different from zero (p<0.05). (DOC) [file pone.0019703.s002.doc]

Table S2: Model fit statistics reporting overall goodness of fit of Equation 2 to the relationship between incubation temperature and the parameters describing the relationship between copper contamination and larval metamorphosis for each species. Values represent the best-fit parameter estimates and their standard errors. All estimated parameter were significantly different from zero (p < 0.05).

| Copper parameter  (Eq 1) | Species | Model R^2^ | Parameter estimate | | |
| --- | --- | --- | --- | --- | --- |
|  |  |  | *M_0_ or C_0_* | *t_M_ or t_C_* | *w_M_ or w_C_* |
| *M_x_* | *A. millepora* | 0.99 | 92 | 33 ± 0.02 | 0.31 ± 0.02 |
| *M_x_* | *A. tenuis* | 0.96 | 88 | 33 ± 0.11 | 0.38 ± 0.12 |
| *EC_50_* | *A. millepora* | 0.98 | 26 | 32 ± 0.15 | 0.78 ± 0.14 |
| *EC_50_* | *A. tenuis* | 0.93 | 32 | 33 ± 0.26 | 0.92 ± 0.25 |
| *w* | *A. millepora* | - | 4.1 ± 1.1 (mean) | | |
| *w* | *A. tenuis* | - | 4.6 ± 0.37 (mean) | | |
